# Successful Management of Aggressive Fibromatosis of the Neck: A Case Report

**DOI:** 10.4274/balkanmedj.2017.0509

**Published:** 2018-05-29

**Authors:** Özgür Mehmet Avinçsal, Hirotaka Shinomiya, Naoki Otsuki, Ryohei Sasaki, Ken-ichi Nibu

**Affiliations:** 1 Department of Otolaryngology-Head and Neck Surgery, Kobe University Graduate School of Medicine, Kobe, Japan; 2 Department of Internal Medicine, Division of Radiation Oncology, Kobe University Graduate School of Medicine, Kobe, Japan

**Keywords:** Aggressive fibromatosis, head and neck, neoplasm, radiotherapy, surgery

## Abstract

**Background:**

Aggressive fibromatoses are histologically benign fibrous neoplasms originating from musculoaponeurotic structures throughout the body. They are locally invasive and erode adjacent vital structures. The head and neck region constitutes 7-25% of all extra-abdominal cases.

**Case Report:**

Here, we report the case of a patient with aggressive fibromatosis in the left side of the neck. While the tumor deeply invaded the scalene muscles, the lesion was successfully treated by surgery followed by radiotherapy. The patient has been disease free for the last 7 years following treatment.

**Conclusion:**

Due to its unusual location in the head and neck region, aggressive fibromatosis should be considered in the differential diagnosis of invading lesions of the neck.

Aggressive fibromatosis (AF) is a nonencapsulated, locally infiltrative, nonmetastasizing tumor. It is histologically benign but has a high rate of local recurrence (1) and can originate from musculoaponeurotic structures in any part of the body. AF is very rare, representing only 0.03% of all neoplasms in humans (2). Its etiology is controversial (1,3). In the head and neck region, AF accounts for 7%-25% of all cases of extra-abdominal fibromatosis (1). Multimodal therapeutic approaches, such as radiation therapy, hormonal therapy, or cytotoxic chemotherapy, are increasingly employed as primary treatments or as adjuvant therapies in incomplete resection or recurrence (4), along with a growing focus on more conservative and individualized strategies. Due to the rarity of AF, particularly in the head and neck region, no clear management guidelines are currently available. Here we report a case of AF in the supraclavicular region. The tumor was treated surgically, preserving vital structures, and by postoperative radiotherapy. Importantly, this is one of few reports including a long follow-up period (7 years).

## CASE PRESENTATION

**Figure 1:**
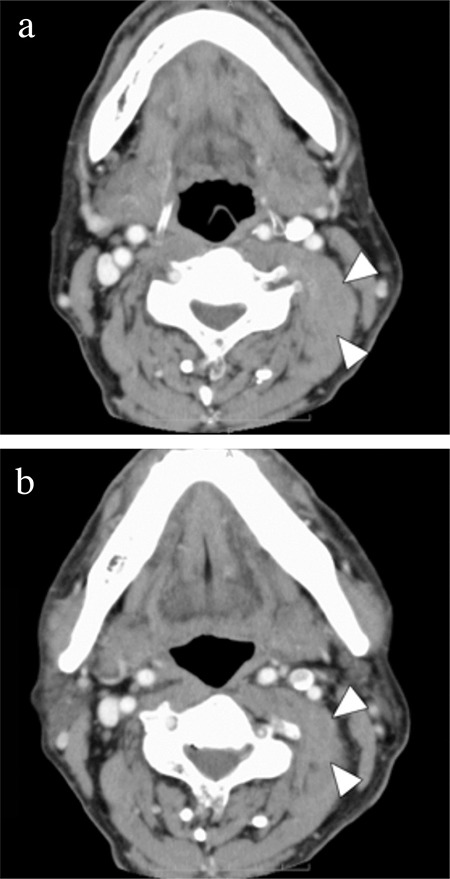
Contrast enhanced-computed tomography findings of left posterior neck mass (a, b). Contrast enhanced-computed tomography scan reveals a large soft tissue mass in the left posterior neck, involving both the anterior and medius scalene muscles (white arrow head).

**Figure 2:**
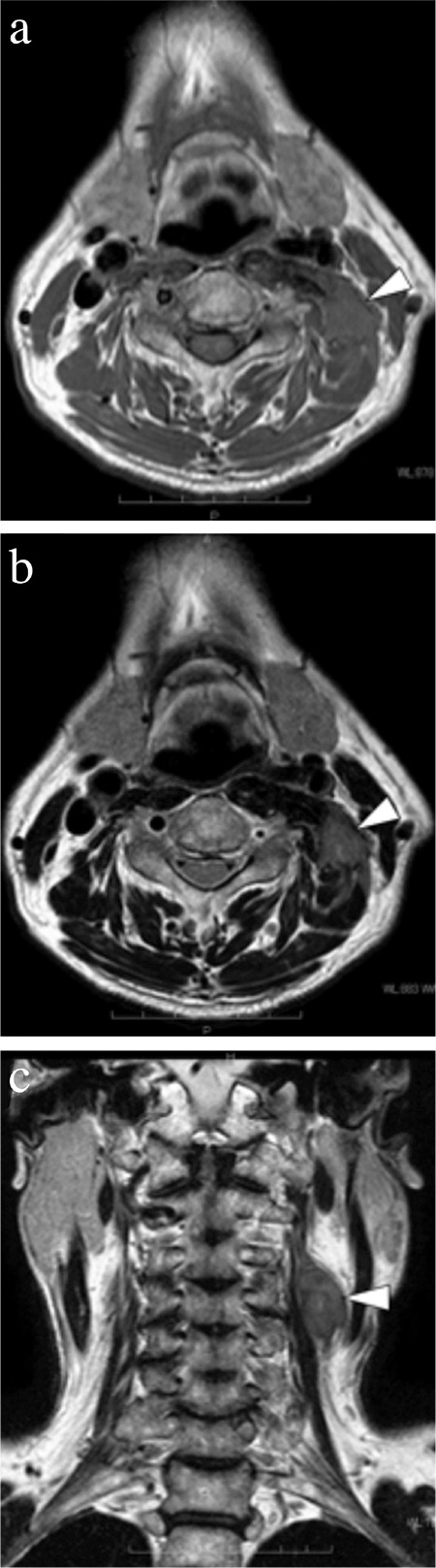
Magnetic resonance imaging of left posterior neck mass (white arrow head). Axial view of T1-weighted (a) and T2-weighted (b), and coronal view of T2-weighted (c).

A 71-year-old male patient was referred to the Department of Otolaryngology-Head and Neck Surgery, Kobe University Hospital, for the evaluation of a 6-month-old, large neck mass that had gradually increased in size. Written informed consent was obtained for surgery including neck dissection. Upon examination, the mass presented as a swelling of approximately 4×5 cm in size, located in the left posterolateral side of the lower neck. It was non-tender, hard, and attached to deep neck structures. A contrast enhanced computed tomography scan revealed a large soft tissue mass in the left posterior neck that involved both the anterior and medius scalene muscles (Figure 1a, b). Magnetic resonance imaging (MRI) revealed an iso-intense mass on the T1 weighted image and a low-intensity mass on the T2 weighted image (Figure 2a-2c). On the anterior side, the mass extended up to the sternocleidomastoid muscle (SCM), with splaying of the left internal jugular vein. No pathological finding was detected in the upper aerodigestive tract by endoscopic examination. After an inconclusive fine needle aspiration, surgical extirpation of the tumor was performed. We did not perform preoperative incisional biopsy because incisional biopsy of neck masses before radical treatment is associated with poor prognosis. The negative influence of preoperative incisional biopsy on prognosis is possibly because of tumor cell dissemination or changes in lymph flow. We were ready to perform a neck dissection procedure if the tumor was malignant. A preoperative incisional biopsy was performed at the beginning of the surgery. The pathological diagnosis of the biopsy was fibromatosis. In response to the clinical findings, a wide excision of the tumor was performed. We encountered difficulty because of local infiltration of the surrounding tissues. Parts of the scalenus medius and SCM were also resected because of tumor invasion. The carotid artery, internal jugular vein, the remainder of the SCM, lower cranial nerves, phrenic nerve, and brachial plexus were successfully preserved. Frozen sections for surgical margins were obtained perioperatively, and the result was negative. On the basis of the intraoperative pathological diagnosis, no additional neck dissection was performed.

**Figure 3:**
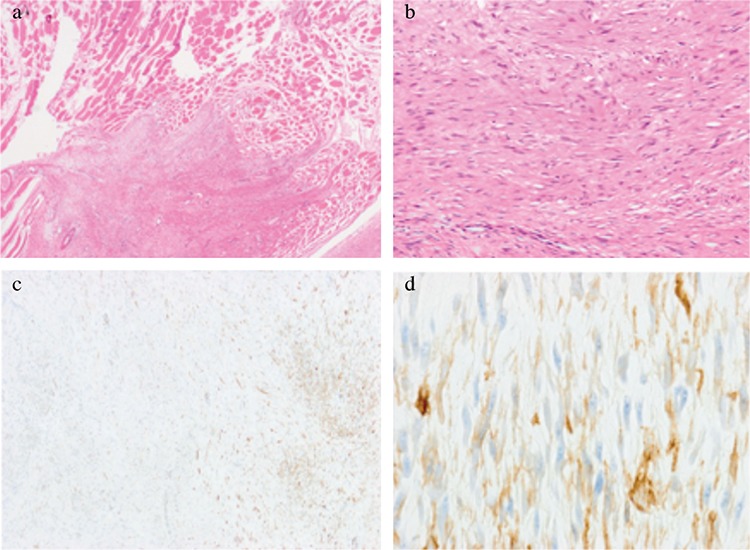
Histopathologic examination of the tumor under low (a, x20) and high (b, x100) power magnification reveals a proliferation of well-differentiated fibroblasts within a collagenous stroma, with infiltration of (H&E stain). Immunohistochemistry shows focally positive cytoplasmic reaction for smooth muscle actin [immune-staining against smooth muscle actin (c, x40), (d, x400)].

Histopathologically, the lesion showed an increase in well-differentiated fibroblasts within a collagenous stroma with infiltration of nearby skeletal muscle and adipose tissues. The excision margins were negative, except for the area adjacent to the scalenus medius muscle. The cells did not show nuclear atypia or hyperchromasia (Figure 3a). Immunohistochemically, the tumor was negative for S-100, beta-catenin, and CD34 but focally positive for alpha-smooth muscle actin (Figure 3b). Based on the clinical behavior and histomorphological features, a definitive diagnosis of AF was made.

**Figure 4:**
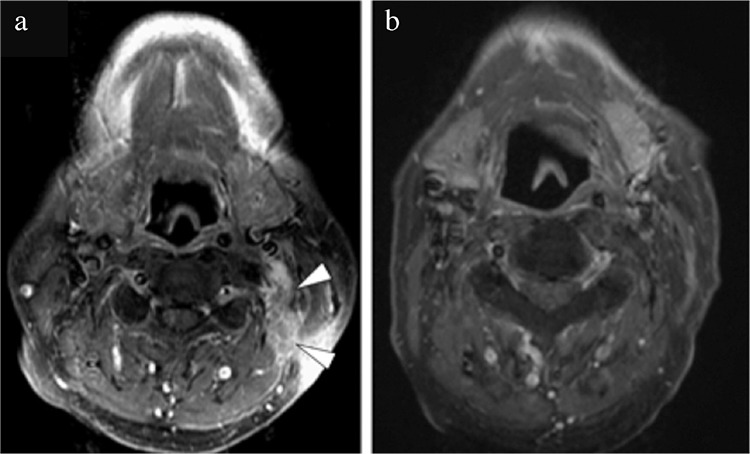
Magnetic resonance imaging finding 3 months after resection (a). A small residual tumor was seen locally above the scalenus medius muscle (white arrow head). The latest magnetic resonance imaging finding (b). No evidence of disease was observed 7 years after radiotherapy.

The postoperative period was uneventful, and the patient recovered completely. Unfortunately, at his 3-month postoperative MRI follow-up exam, a small residual tumor was detected in the scalenus medius muscle (Figure 4a). The recurrent lesion was treated by radiotherapy with a total dose of 70 Gy/35 Fr, resulting in significant regression. The patient has been on close, regular follow-up for the last 7 years at our clinic. Radiographic studies have shown no evidence of tumor recurrence (Figure 4b).

## DISCUSSION

AF tends to be associated with significant morbidity when it occurs in the head and neck region, due to the infiltration or proximity to vital anatomic structures and a tendency for local recurrence. According to the literature, wide surgical resection with clear margins is the most common first-line treatment for AF (5). Nevertheless, its clinical course can be variable and unpredictable. In some cases, the tumor recurs and grows rapidly even after wide surgical resection; however, in other cases, the tumor remains stable for long periods and even regresses spontaneously (6). Several studies have shown that positive margins are associated with a higher recurrence rate (2,7), although other studies do not consider this to be a statistically significant prognostic factor (5). Therefore, function-preserving surgery should be the major goal to minimize morbidity. If tumor-free margins cannot be achieved for cosmetic or functional reasons, postoperative radiotherapy should be considered as adjuvant therapy to improve local control (8). In our patient, the tumor was attached to the supraclavicular fossa and adherent to the scalene muscles, expanding nearby to involve vital anatomic structures, such as the internal jugular vein and accessory and phrenic nerves. Nonetheless, the tumor could be excised en bloc and all vital structures were preserved. However, unfortunately, the excision margins adjacent to the scalenus medius muscle were positive. Given the lack of clear guidelines, surgeons should refer to the best evidence available, the individual patient’s wishes, and the morbidity of the treatment. Considering all these factors in the present case, we decided on close clinical and radiological follow-up after surgery. Owing to the rarity of the tumor, the prognostic factors for the local recurrence of AF are uncertain. Nevertheless, a close or positive margin was found to be a risk factor for recurrence (5). Although attempts to achieve negative margins may cause additional morbidity, the extent of adequate surgical margins has not been defined yet. Several studies suggested variable tumor-free margins, within variable distances (1-10 mm). Therefore, the minimum distance for the surgical resection margins required to reduce the possibility of local recurrence is still unknown. AF tumors consist of proliferating spindle-shaped, slender, or elongated cells in a dense keloid-like collagen stroma. Despite its classification as a histologically benign neoplasm without metastatic potential, AF is poorly encapsulated and locally invasive; therefore, complete surgical excision is difficult and recurrences are frequent. The rate of local recurrence after surgery of AF in the head and neck is typically between 24% and 70% (3). Recurrences in the head and neck generally arise in the first 2 years, but they may occur anytime from a few months to >10 years after the initial surgery (4). Therefore, close lifelong follow-up of a patient with AF is essential. For disease extension investigation and follow-up examinations, MRI is the imaging modality of choice because it provides better soft-tissue definition than computed tomography (9). Typical AF tumors are isointense on T1-weighted images and heterogeneously hyperintense on T2-weighted images (9). Our patient is still under close follow-up and has had no local recurrences in the past 7 years.

Adjuvant treatment options include radiotherapy; a wait-and-see policy; and administration of tamoxifen, non-steroidal anti-inflammatory drugs (NSAIDs), and cytotoxic drugs. Hormone therapy may have a significant role, considering the possible hormonal etiopathogenesis of the condition. One of the most commonly tried chemotherapeutic drugs is the antiestrogenic drug tamoxifen (10). However, the results of this type of treatment have not yet been fully analyzed. NSAIDs may also have a role in treatment. Indomethacin, sulindac, and colchicine have been tested in patients with AF with varying degrees of success (10). In the event that anti-estrogen therapy and NSAIDs fail to act sufficiently, cytotoxic drugs, such as doxorubicin-dacarbazine or doxorubicin-cyclophosphamide-vincristine combinations or a vincristine, actinomycin-D, cyclophosphamide regimen, may be used (10). Our patient received radiotherapy as the adjuvant treatment option. Given the rarity of AF in the head and neck region, it should be considered in the differential diagnosis of masses in the neck. Importantly, the rarity of AF presents challenges to the planning of treatment and patient follow-up.
